# A high-quality *Actinidia chinensis* (kiwifruit) genome

**DOI:** 10.1038/s41438-019-0202-y

**Published:** 2019-10-15

**Authors:** Haolin Wu, Tao Ma, Minghui Kang, Fandi Ai, Junlin Zhang, Guanyong Dong, Jianquan Liu

**Affiliations:** 1 Key Laboratory of Bio-Resource and Eco-Environment of Ministry of Education and State Key Laboratory of Hydraulics and Mountain River Engineering, College of Life Sciences, Sichuan University, Chengdu, 610065 China; 2 The Limited Agriculture Company of Xinyuan Sacred Fruit, Shifang, Deyang, 618409 Sichuan China; 3 State Key Laboratory of Grassland Agro-Ecosystem, Institute of Innovation Ecology, Lanzhou University, Lanzhou, 730000 China

**Keywords:** Comparative genomics, Genome evolution

## Abstract

*Actinidia chinensis* (kiwifruit) is a perennial horticultural crop species of the Actinidiaceae family with high nutritional and economic value. Two versions of the *A. chinensis* genomes have been previously assembled, based mainly on relatively short reads. Here, we report an improved chromosome-level reference genome of *A. chinensis* (v3.0), based mainly on PacBio long reads and Hi-C data. The high-quality assembled genome is 653 Mb long, with 0.76% heterozygosity. At least 43% of the genome consists of repetitive sequences, and the most abundant long terminal repeats were further identified and account for 23.38% of our novel genome. It has clear improvements in contiguity, accuracy, and gene annotation over the two previous versions and contains 40,464 annotated protein-coding genes, of which 94.41% are functionally annotated. Moreover, further analyses of genetic collinearity revealed that the kiwifruit genome has undergone two whole-genome duplications: one affecting all Ericales families near the K-T extinction event and a recent genus-specific duplication. The reference genome presented here will be highly useful for further molecular elucidation of diverse traits and for the breeding of this horticultural crop, as well as evolutionary studies with related taxa.

## Introduction

Kiwifruit (known as “the king of fruits” because of its remarkably high concentration of vitamins, minerals, and other nutrients) is produced by various species of the genus *Actinidia* (Actinidiaceae). Total estimated annual sales exceed 10 billion dollars globally. More than 50 species of the genus have been described, and more than 60 cultivars are grown throughout the world^1^. Major cultivars have been domesticated or bred from *Actinidia chinensis* Planchon, with ploidy levels ranging from diploid (2*n* = 2× = 58) to octoploid. A diploid cultivar of this species was selected to assemble genomes in the present and previous studies. The first released *A. chinensis* genome^2^ (hereafter v1.0) was assembled from short Illumina reads, and the second (of genotype Red5^3^, hereafter v2.0) was assembled from short Roche 454 reads. They provide valuable information but require improvement because of difficulties in assembling the short reads into long contigs and scaffolds. Use of SMRT (single molecule real-time) sequencing technologies, PacBio sequencing, for example, can generate long reads, up to ~30–40 kb. This has enabled the generation of greatly improved and updated genomes of numerous crops (including apple^4^, rice^5,6^, broomcorn millet^7^, tea tree^8^, and durian^9^), with several orders of magnitude greater continuity than previous versions (increases in N50 from tens of kilobases to >1 Mb). Moreover, most generated scaffolds can be anchored into chromosomes based on interaction frequencies of sequences (Hi-C data), thus providing high-resolution chromosome-level assemblies.

Here, we report such a high-quality de novo chromosome-level genome for a heterozygous kiwifruit, ‘Hongyang’ (hereafter *A. chinensis* genome v3.0), which is widely grown in China; the genome was generated by assembling long PacBio reads and mapping scaffolds based on Hi-C interaction confirmation. Using this high-quality genome, we further examined whether the two whole-genome duplications suggested for the kiwifruit genome^10^ also occurred in closely related groups. This reference genome provides valuable foundations for agronomic understanding and molecular breeding of kiwifruit in the future, including cloning key genes to control fruit traits and identifying disease-resistance alleles. Such a high-quality genome is also highly beneficial for exploring genome evolution and genetic change underlying species diversification of the Actinidiaceae and closely related families of the Ericales.

## Results

### Sequencing and assembly of a new de novo genome

We used one female of the Chinese kiwifruit cultivar ‘Hongyang’ for whole-genome sequencing and applied a hierarchical approach for chromosome-scale assembly. We first produced ~30.16 Gb Illumina paired-end short reads (270 bp) and used them to estimate the sampled individual’s heterozygosity ratio (0.76%). We also obtained an estimate of the genome size (637.99 Mb) from the 19-mer depth distribution of the Illumina reads (Supplementary Fig. 1), substantially smaller than previous estimates obtained using the preQC and Jellyfish tools (705 Mb and 142 Mb, respectively)^3^.

We then generated ~41.09 Gb of raw PacBio reads (providing ~40-fold genome coverage, N50 = 12.11 kb) and assembled them into 2231 contigs using SMRT sequencing technology, finally producing a 653.86 Mb genome with a longest contig of 9.96 Mb and an N50 contig length of 1.72 Mb (Supplementary Table 1). We used three data sources to evaluate the quality of the *A. chinensis* genome v3.0. First, we aligned our Illumina and RNA-seq reads to the v3.0 assembly using BWA^11^, HISAT2^12^ and Blasr^13^, which showed good alignments with proper mapping rates of 92.47% and 91.00%, respectively (Supplementary Tables 2 and 3). Next, we searched for core eukaryotic genes (CEGs) and found 427 of 458 in the newly assembled genome using CEGMA14 (Supplementary Table 4), suggesting that most of the CEGs were complete in our newly assembled genome. Moreover, as shown in Supplementary Table 5, in a further test of our assembly’s integrity, we found that 90.8% of our gene set consisted of complete single-copy Benchmarking Universal Single-Copy Orthologs (BUSCOs)^14^. These results collectively suggest that our new genome is well assembled with high quality.

**Fig. 1:**
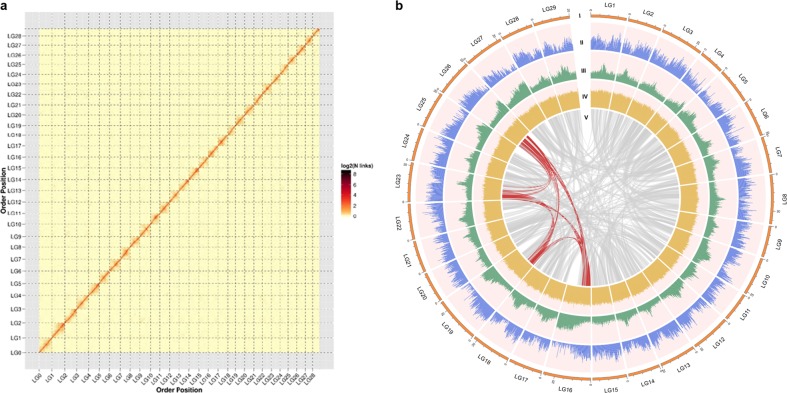
Hi-C assisted assembly of *A. chinensis* v3.0 pseudomolecules. **a** Heatmap showing Hi-C interactions under a resolution of 200 kb, and the antidiagonal pattern for the intrachromosomal interactions may reflect the Rabl configuration of chromatins. **b** The landscape of genome assembly and annotation of *A. chinensis* v3.0. Tracks from outside to the inner correspond to I, pseudomolecules; II, gene density; III, repeat density; IV, GC content; and V, synteny information

We further generated 75.96 Gb Hi-C clean data with ~116 × coverage (Supplementary Table 6). We anchored 98% of the assembly (640.75 Mb) onto 29 pseudochromosomes and oriented 91.01% of the assembly (583.11 Mb) with the aid of the Hi-C sequence data using a hierarchical clustering strategy^15^ (Fig. 1; Supplementary Fig. 3; Supplementary Tables 7 and 8). Our assembly v3.0 is larger than both previous genome versions (Table 1).

**Table 1:** A comparison of the three published *A. chinensis* genomes

Assembly feature	v1.0	v2.0	v3.0
Number of scaffolds	7698	3887	2366
Scaffold N50	646.8 kb	623.8 kb	1.43 Mb
Longest scaffold	3.41 Mb	4.43 Mb	7.81 Mb
Assembly length	616.1 Mb	550.5 Mb	653.9 Mb
Repeat region % of assembly	36%	38.28%	43.42%
Predicted gene models	39,761	33,044	40,464
Average CDS length	1103 bp	1047 bp	1028 bp
Exons per gene	4.49	4.82	5.47

### Contiguity and accuracy improvement

Our *A. chinensis* v3.0 assembly provides substantial improvements (with fewer gaps and larger contig sizes) than the v1.0 and 2.0 genomes, which were assembled from shorter Illumina and 454 reads, respectively. Both previous versions have limitations because of the frequent fragmentation and low contiguity (Table 1). The contig N50 value of the new assembly, obtained by combining PacBio long reads and Hi-C data, is 1820 kb, which is ~2.9-fold and ~2.8-fold greater than the corresponding values for v1.0 and 2.0 (646.8 kb and 623.8 kb, respectively; Table 1). Moreover, only 646 gaps were found in v3.0, and its contiguity (0.1 kb) is ~365-fold and ~448-fold better than those of the previous versions (44.1 kb and 35.9 kb, respectively; Supplementary Tables 8 and 9). Accordingly, there are ~47-fold and ~44-fold fewer gaps/Mb in v3.0 than in v1.0 and v2.0 (43.36 and 46.36, respectively; Supplementary Tables 8 and 9).

We also assessed the scaffold orderings of the three *A. chinensis* genomes through a series of whole-genome sequence alignments (Supplementary Fig. 4). Despite strong overall collinear relationships among the three genomes, we found that our v3.0 assembly has more assembled regions than the other two, especially v1.0. For example, LG10 in v3.0 is not covered by v1.0 (Supplementary Fig. 4a). These regions, containing abundant repeated sequences, are completely covered by the long PacBio reads but not by the short Illumina reads. Furthermore, the new genome illuminates inconsistencies between the v1.0 and v2.0 assemblies. For example, an 8.1 Mb region mapped to LG19 in v1.0 maps to LG23 and LG29 in v2.0 (Supplementary Fig. 4b), and our results support v1.0 (Supplementary Fig. 4a). We also compared the v3.0 genome with a recently published chromosome-level genome of *A. eriantha* and found that they are highly collinear with no clear chromosomal or structural differences^16^ (Supplementary Figs. 4c and 5). Taken together, all the comparisons and independent validations suggest that v3.0 has better contiguity and ordering of contigs than the other two *A. chinensis* assemblies, suggesting that this newly assembled genome has high quality.

### Annotation of repeat sequences

Using homology-based and de novo approaches to identify transposable elements, we identified repeat regions spanning ~270.8 Mb (43.42% of the assembly size) in our v3.0 genome, substantially more than in the previous two genomes (36%, spanning 222 Mb in v1.0 and 38.28% spanning 202 Mb in v2.0; Table 1). This indicates that the PacBio data facilitated the construction of repetitive regions (Fig. 1).

Most repetitive elements in our novel assembly are long terminal repeat (LTR) retrotransposons, especially Gypsy-LTRs and Copia-LTRs. These sequences, detected by LTR_retriever^17^, span 23.38% of the assembled genome, substantially more than identified in v1.0 (152.28 Mb, 24.72%) and v2.0 (102.06 Mb, 18.65%) (Supplementary Table 10). We also identified 1246 intact LTR-RTs in v3.0, compared to just 522 and 856 in v1.0 and v2.0, respectively (Supplementary Table 11 and Supplementary Fig. 6). We found 1212 intact LTR-RTs (97.27%) in v3.0, again substantially more than in v1.0 and v2.0 (620 and 521, respectively; Supplementary Table 11). The estimated insertion times of the intact LTR-RTs indicate that expansions occurred recently, less than one million years ago (Ma) (Supplementary Fig. 7).

Using the standardized metric LTR Assembly Index, based on LTR-RTs, we evaluated the quality of assembled intergenic and repetitive sequences^18^ in the three assembled genomes and obtained higher scores for v3.0 than for both v1.0 and v2.0 (Supplementary Fig. 8). This confirms the significant improvement in assembly continuity afforded by using long PacBio reads rather than shorter Illumina or 454 reads (Fig. 1).

### Gene prediction and annotation

Using a combination of homology-based transcript-alignment and de novo methods, we predicted 40,464 protein-encoding genes in the v3.0 genome. The coding sequences of the predicted genes are 1028 bp long and have 5.5 exons, on average (Table 1). We used the Circos tool (http://www.circos.ca) to map GC content, repeat density and gene density of each chromosome (Fig. 1b), and validated functions of the predicted genes. We detected genes with sequences meeting standard homology criteria for 71.51% and 85.60% of the genes in the Swiss-Prot and TrEMBL databases^19^, respectively. In addition, we successfully annotated 93.55, 81.82, and 24.35% of the genes using the InterPro^20^, GO^21^ and KEGG^22^ Pathway databases, respectively. Overall, we assigned potential functions to 38,202 of the 40,464 protein-coding genes (94.41%) in the *A. chinensis* v3.0 genome (Supplementary Table 12; Supplementary Fig. 9).

The number of annotated genes in assembly v3.0 (40,464) is higher than the numbers in v1.0 (39761) and v2.0 (33,044) (Table 1). Moreover, 90.16% (36,482 of 40,464) of the genes are located on chromosomes, compared to just 31,409 (79.00%) and 32,951 (99.72%) in v1.0 and v2.0, respectively (Supplementary Table 8). We further evaluated the quality of the annotation using the BUSCO set of 1440 highly conserved genes in plants and identified ~89.1% BUSCOs in v3.0, more than in v1.0 (76.6%) although less (following manual adjustment) than in v2.0 (94.5%) (Supplementary Table 13).

**Fig. 2:**
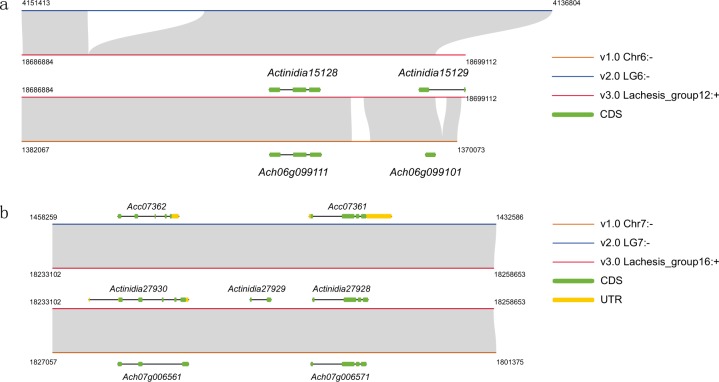
Examples showing invalid or missing gene annotations in v1.0 and v2.0. **a** An example of a region (including repeat sequences) that exists in v3.0 but cannot be assembled in v1.0, resulting in a truncated annotation of the gene *Ach06g099101*, indicating an invalid annotation in v1.0 and a missing annotation in v2.0. **b** Two genes, *Actinidia27930* and *Actinidia27928*, in v3.0 are roughly annotated in v2.0 (*Acc07362* and *Acc07361*) and v1.0 (*Ach07g006561* and *Ach07g00657*), but a third one between them, *Actinidia27929*, could not be recovered in either v2.0 or v1.0

A total of 2779 and 4365 genes with obvious gaps in v1.0 and v2.0 were rectified in the annotated genes of *A. chinensis* genome v3.0 (Supplementary Table 14). Furthermore, we identified 2750 specific genes in v3.0, compared to 2205 in v1.0 and 575 in v2.0 (Supplementary Table 14). Among the version-specific genes in the newly assembled genome, 62.8% (1727 of 2750) were confirmed by functional validation. In addition, because more repetitive sequences were annotated in v3.0, the annotated genes covered regions that were not assembled in the previous versions. For example, some genes in v1.0 were shorter than the corresponding genes in v3.0 (Fig. 2a), possibly due to a failure to assemble repeat regions using the short Illumina reads in v1.0. We also mapped the reads used to assemble the two previous versions to our genome v3.0 to verify the accuracy of the corresponding genes in v3.0 (Supplementary Fig. 10). We found that some gene reads had not been annotated, although they were present in both the v1.0 and v2.0 genomes (Fig. 2b). All of these corrected genes in v3.0 could be functionally annotated through reference to entries for orthologous *Arabidopsis thaliana* genes in the Gene ontology (GO), Swiss-Prot, TrEMBL, and InterPro databases.

### Whole-genome duplications

**Fig. 3:**
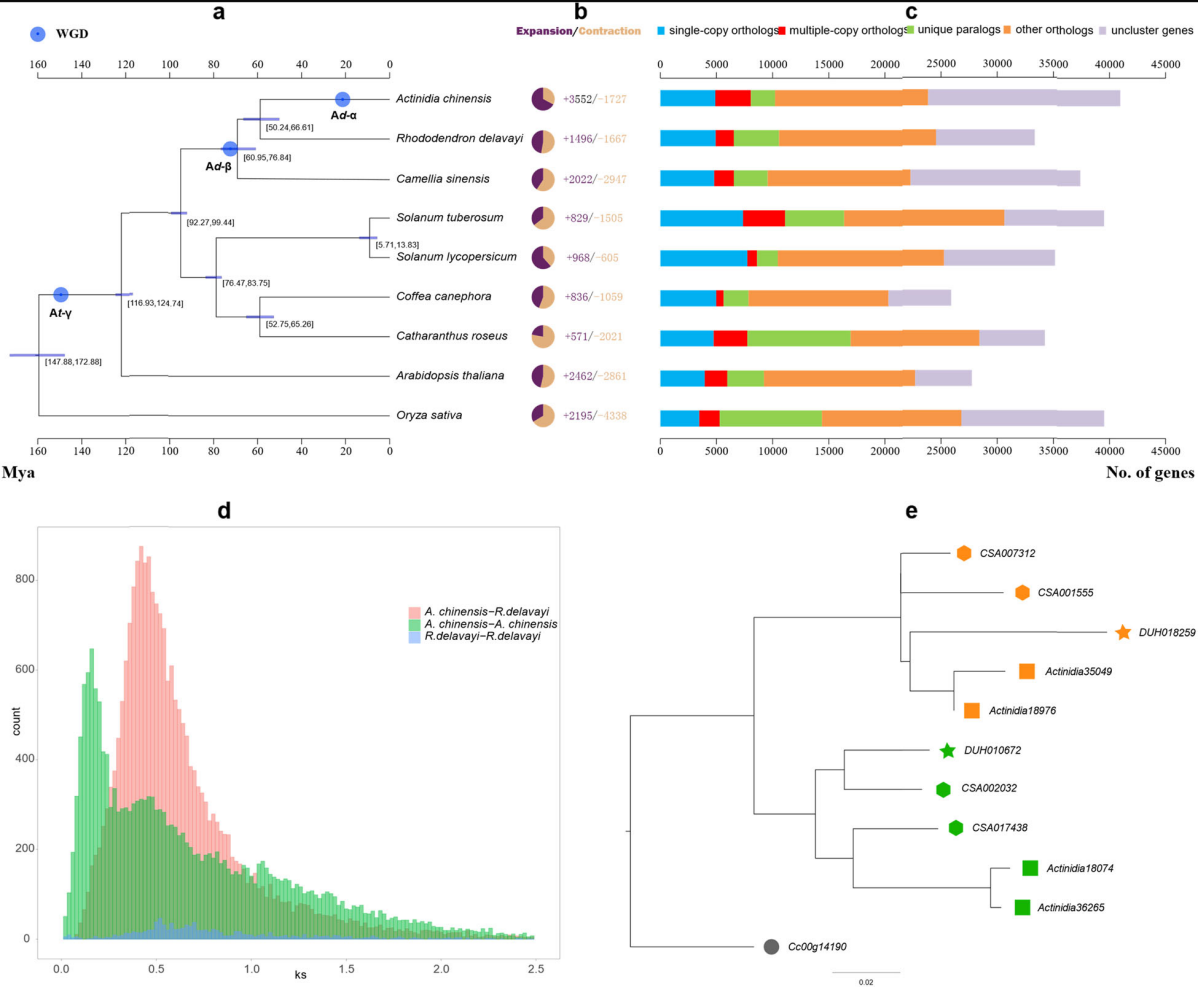
Phylogenetic and evolutionary analyses of the *A. chinensis* genome v3.0. **a** Phylogenetic relationships and divergence times between *A. chinensis* and other plant species. The blue circles represent three WGD events in the evolutionary history of *A. chinensis*. **b** Expansions and contractions of gene families. The colors in purple and yellow indicate the expanded and contracted gene families, respectively. **c** Clusters of orthologous and paralogous gene families in kiwifruit and eight more fully sequenced plant genomes. Only the longest isoform for each gene was used. Gene families were identified using the OrthoMCL package with the default parameters. **d** Distribution of the synonymous substitution rate (Ks) between *A. chinensis* and *R. delavayi*. **e** A phylogenetic tree of the homologous genes from *Actinidia* (rectangle), *Rhododendron* (star), *Camellia* (hexagon), and *Coffea* (circle), supporting the shared A*d*-β event in the Ericales. Homologous genes belonging to the A*d*-α and A*d*-β events are shown in orange and green, respectively

We clustered the annotated *A. chinensis* genes with those of *Oryza sativa*, *Arabidopsis thaliana*, *Catharanthus roseus*, *Coffea canephora*, *Solanum lycopersicum*, *Solanum tuberosum*, *Camellia sinensis,* and *Rhododendron delavayi* by OrthoMCL^23^. Of the 40,464 protein-coding genes in genome v3.0, 23,560 genes were grouped into 15,294 gene families with an average of 1.54 genes per family, and 875 gene families were kiwifruit-specific (Fig. 3c; Supplementary Table 15). We used 1517 single-copy orthologous genes identified in nine species for phylogenetic analysis based on the maximum likelihood method. The results indicate that *A. chinensis* is most closely related to *R. delavayi* (Fig. 3a). Using MCMCtree^24^ with fossil calibration, these two species were estimated to have diverged 50.2–66.6 Ma (Fig. 3a), as previously suggested^25^. Furthermore, using CAFÉ^26^, we found indications that 3552 gene families had expanded and 1727 contracted in our newly assembled kiwifruit genome (Fig. 3b).

Whole-genome duplication (WGD) or polyploidization appears to have occurred in most plant species’ evolutionary histories^27^. Using the MCScanX^28^ package, we identified 2041 syntenic gene blocks containing 22,689 collinear gene pairs in the *A. chinensis* and *R. delavayi* genomes. The calculated synonymous substitution rates (*Ks*) of these gene pairs peaked at 0.38 to 0.44, suggesting that the *A. chinensis* and *R. delavayi* genomes diverged from their last common ancestor at ~50.24 to 66.61 Ma, based on a universal substitution rate of 3.39 × 10^−9^ mutations per site per year (Fig. 3d). Moreover, we identified 22,165 paralogous gene pairs in the newly assembled genome, accounting for 56% of the protein-coding genes. Based on these duplicated gene pairs, we calculated an age distribution of *Ks* with peaks of ~0.12–0.18, 0.42–0.5, and 1.02–1.08 (Fig. 3d; Supplementary Table 16), corresponding to three putative WGD events (A*d*-α,17.7–26.5 Ma; A*d*-β, 61.9–73.7 Ma and A*t*-γ, 150.4–159.3 Ma) in the kiwifruit genome’s evolutionary history. The earliest WGD (A*t*-γ) was confirmed to be shared by all eudicots.

The two recent WGDs (A*d*-α and A*d*-β) were also suggested by a recent study^10^, within similar timeframes, depending on the applied calibrations or mutation rates. We also examined whether these two recent WGDs occurred in the closely related *R. delavayi* genome and found that only one recent WGD occurred in it, at a similar date to A*d*-β of *A. chinensis* (Fig. 3d). However, this WGD stage partly overlapped with divergence between *A. chinensis* and *R. delavayi* (Fig. 3). A WGD was also detected within this timeframe, in addition to a more recent WGD in the genome of the tea family^29^, which is closely related to *A. chinensis* and *R. delavayi*.

We further distinguished whether the three species representing three families shared the same WGD(s) or lineage-specific WGDs shortly after their divergences during the Cretaceous-Tertiary (K-T) boundary, when WGDs frequently occurred in plants^30^. Following previously published procedures^10^, we first constructed phylogenetic trees of the homologous genes. As no additional WGD has been reported for coffee since the ancient A*t*-γ WGD, we extracted one, four, two and four sets of homologous genes from the *Coffea*, *Camellia*, *Rhododendron,* and *Actinidia* genomes, respectively. We recovered only one homologous group, for which we constructed a gene tree and found two well-supported lineages for *Actinidia*, *Camellia*, and *Rhododendron*, suggesting they shared a WGD (Fig. 3e). We also found that in both *Camellia* and *Actinidia*, each of the two separate subgroups evolved into two sets of genes. This supports the hypothesis that two recent WGDs occurred independently in *Camellia* and *Actinidia*, in addition to the common earlier WGD, although the two gene sets from *Camellia* did not cluster together, possibly due to annotation errors (Fig. 3e).

The duplicated genes may have been randomly lost in the descendent lineages after the earlier WGD, with consequent difficulties in identifying all paralogous genes in different lineages. Therefore, we used only *Coffea* and two other randomly selected genomes to examine whether they shared WGDs. For this, we extracted sets of three homologous gene groups: *Coffea* (1): *Camellia* (4): *Actinidia* (4); *Coffea* (1): *Rhododendron* (2): *Actinidia* (4) and *Coffea* (1): *Camellia* (4): *Rhododendron* (2). In total, we recovered 1, 8, and 55 homologous groups, respectively. We successfully constructed gene trees for 1 (100%), 2 (25%), and 24 (44%) of these homologous groups, respectively (Supplementary Fig. 11), and discarded the other groups due to low statistical support. These trees also supported the common WGD for all three families of the Ericales and two independent WGDs for *Actinidia* and *Camellia*. However, both *Camellia* and *Rhododendron* genomes require improvement because numerous homologous genes from them could not be correctly identified and analyzed. Thus, all inferences presented here can potentially be further validated in the future following more accurate annotation of their genes.

## Discussion

Here, we describe a third de novo assembly of the *A. chinensis* genome obtained by integrating data from PacBio, Hi-C, and Illumina platforms. Our use of PacBio sequencing led to a clear improvement in contig N50 size, which filled many gaps and corrected many misassemblies in the previous two versions (Table 1; Supplementary Table 8). Our scaffolding with Hi-C further facilitated the accurate assignment of all scaffolds to chromosomal positions. This new assembly has higher genome contiguity and sequence quality than the previously published in twos. It also includes substantially more TEs (especially intact LTR-RTs). The results of further analyses suggest that most LTR-RT expansions occurred in the last million years. Our assembly also includes more annotated genes than v1.0 and v2.0 and more assembled regions (>100 Mb, mainly repeating regions) than v2.0, which undoubtedly facilitated the more complete and accurate gene annotation. The newly available genic resources will facilitate agronomic dissection of traits of this important fruit and facilitate its breeding at the molecular level. The genome will be particularly useful for identifying disease-resistant alleles.

WGDs occurred frequently during the K-T boundary, when most plant lineages started to diversify greatly^27^. During this period, numerous species became extinct on Earth (for example, all dinosaurs), an event known as the K-T extinction. WGDs may have been essential for lineages’ survival and subsequent explosive radiations^27^ during this event, or this event may have triggered WGD(s) in the lineages that survived and explosively speciated. Ericales is a species-rich order, with more than 8000 species, but it is unclear whether all ericaceous families experienced a common WGD near the K-T extinction event and/or independent WGD(s) occurred after their divergences. Analyses with our new *A. chinensis* genome v3.0 indicate that this species shared a K-T WGD with members of both Ericaceae and Theaceae, in addition to more recent genus-specific WGD(s) that have occurred in both the *Camellia* and *Actinidia* genomes. All these results are highly beneficial for our understanding of the Ericales’ survival and diversification history.

## Material and methods

### DNA extraction and sequencing

We extracted high-quality genomic DNA from fresh young leaves of a cultivated diploid *A. chinensis* female (cv. Hongyang) plant growing on a farm in Shifang, Sichuan Province, China, using a DNAsecure Plant Kit (Tiangen Biotech, Co. Ltd, Beijing, China). For Illumina sequencing, we constructed a paired-end library with insert sizes of 270 bp and subsequently sequenced the reads using an Illumina HiSeq X Ten platform. This yielded a total of 30.16 Gb clean Illumina data (Supplementary Table 1). For PacBio sequencing, we fragmented, digested, and ligated the total genomic DNA, as suggested by Pacific Biosciences. We prepared 20-kb SMRTbell libraries and sequenced them using a PacBio Sequel sequencer and then used the PacBio SMRT-Analysis package to process raw reads with readScore = 0.75 and minSubReadLength = 500. We removed sequencing adapters and filtered reads with low quality and short lengths. This yielded 41.09 Gb clean reads with N50 length = 12.11 kb (Table S1).

### Genome assembly

For genome assembly, we first corrected potential errors in the PacBio reads by Canu^31^ (v1.5) and then used Falcon v0.7^32^, WTDBG v1.2.8 (https://github.com/ruanjue/wtdbg), and Canu v1.5 to independently assemble the high-quality PacBio subreads. These packages yielded 1.02 Gb, 896.96 Mb, and 642.21 Mb assemblies, with N50 values of 161.14 kb, 187.27 kb, and 866.55 kb, respectively. The homologous contigs were further optimized and corrected based on self-alignment and depth. The well-assembled Canu and WTGDB results were merged by Quickmerge^33^. The merged genome was corrected with the Illumina data using Pilon. The final assembled genome size is 653.86 Mb.

To evaluate the assembled genome’s quality, we first mapped the Illumina NGS and PacBio data to it using BWA-mem^11^ and Blasr^13^, respectively, then mapped core eukaryotic genes (CEGs) using CEGMA^34^, and finally applied a BUSCO test^14^, with the Embryophyta_odb9 database and default parameters, to examine its gene content.

### Chromosome assembly using Hi-C

A sample of ~2 g of fresh young leaves of the same kiwifruit accession was ground in liquid nitrogen for the Hi-C experiment. We fixed the leaves with formaldehyde and digested the cross-linked DNAs with HindIII, then biotinylated the sticky ends and proximity-ligated them to form chimeric junctions. We enriched these junctions and physically sheared them to a size of 300–700 bp. We constructed sequencing libraries using NEBNext Ultra enzymes and Illumina-compatible adapters. We isolated the biotin-containing fragments using streptavidin beads and then PCR-enriched each library. We examined the insert sizes using Qubit 2.0 and Agilent 2100. We accurately quantified the effective concentration of the libraries by Q-PCR to confirm their quality and then sequenced them using an Illumina HiSeq platform. We obtained a total of 75.96 Gb clean Hi-C data, providing ∼116-fold coverage of the *A. chinensis* genome, which we used for chromosome-level assemblies, via the pipeline shown in Supplementary Fig. 2. In a preassembly step, to correct errors in scaffolds, scaffolds were split into segments of 200 kb on average. We then mapped the Hi-C data to the segments using BWA (v0.7.10-r789) software^11^. We retained the uniquely mapped data for assembly using LACHESIS software^15^ with parameters CLUSTER_N = 10, CLUSTER_MIN_RE_SITES = 48, ORDER_MIN_N_RES_IN_TRUN = 14, CLUSTER_MAX_LINK_DENSITY = 2, CLUSTER_NONINFORMATIVE_RATIO = 2, ORDER_MIN_RES_IN_SHREDS = 15. Detailed information on the Hi-C read mapping is presented in Supplementary Table 6. We also removed duplicates and assessed quality with HiC-Pro (v2.8.1)^35^. We then assembled the corrected contigs with LACHESIS and then visualized the interaction matrix of all chromosomes and heatmaps with a resolution set at 200 kb. In total, we anchored 675 contigs (representing 98% of the total length) to 29 chromosomes in the *A. chinensis* genome assembly.

### Whole-genome alignment of *A. chinensis genomes*

We aligned our v3.0 genome sequence to the previously published genomes of the species by LAST^36^, following a published five-step procedure (https://github.com/mcfrith/last-genome-alignments) and then verified the alignments using in-house Perl scripts based on the condition of the three pairs of alignments.

### Identification of repetitive elements

We identified repetitive elements by a combination of homology alignment and de novo searches, as follows. We used RepeatMasker^37^ with Repbase (v.16.10)^38^ to scan for sequences homologous to annotated repeat sequences in published databases and then used RepeatModeler^38^ (http://www.repeatmasker.org/RepeatModeler.html) with the default parameters to predict de novo transposable elements (TEs). We combined the repeat sequences identified by both methods as the final annotated repeat set. We integrated the overlapping transposable elements and removed those with low scores.

We identified candidate LTR-RTs using LTR_Finder (v1.02)^39^ (parameters: -D 15000 -d 1000 -L 7000 -l 100 -p 20 -C -M 0.9) and LTRharvest^40^ (parameters: -similar 90 -vic 10 -seed 20 -seqids yes -minlenltr 100 -maxlenltr 7000 -mintsd 4 -maxtsd 6 -motif TGCA -motifmis 1). We integrated these results and discarded false positives by the LTR_retriever pipeline^17^ and then estimated insertion times based on *T* = *K*/2*r*, where *K* is the divergence rate and *r* is the neutral mutation rate (3.39 × 10^−9^). Raw LAI was estimated as ‘intact LTR element length/total LTR sequence length) × 100’. We also obtained raw LAI scores (by dividing the intact LTR element length by the total LTR sequence length and multiplying by 100) using the LAI program of the LTR retriever package, with window size and sliding step set to 3 Mb and 300 kb, respectively^18^.

### Gene prediction

The homology-based, transcriptome-based, and ab initio predictions were integrated to predict the high-quality protein-coding genes. During the homology-based prediction, the protein sequences of *A. thaliana*, *O. sativa*, *S. lycopersicum*, *V. vinifera*, *C. sinensis*, *R. delavayi,* and *A. chinensis* cv. Red5 from NCBI or Phytozome (v12.1; https://phytozome.jgi.doe.gov/) were downloaded and aligned to the genome using TBLASTN (*E*-value threshold 1 × 10^−5^). We aligned homologous genomic sequences against matching proteins to define gene models using GeneWise^41^. For prediction based on the RNA-seq data, we downloaded all RNA-seq data from the NCBI database (NCBI SRA accessions: SRR653044, SRR653045, SRR653046, SRR653047, SRR5650770) of this species. All RNA reads were initially aligned based on the reference genome by HISAT2^12^ and further assembled into transcripts using Trinity (v2.6.6)^42^. These assembled sequences and full-length transcripts obtained by PacBio sequencing were aligned to the *A. chinensis* genome by Program to Assemble Spliced Alignment (PASA)^43^. We then clustered the valid transcript alignments according to the locations obtained from genome mapping and finally assembled them into gene structures. For ab initio prediction, Augustus^44^ was run on the genome with parameters trained using PASA self-trained gene models. The gene models obtained using the three approaches were merged as the final consensus set using EVidenceModeler (EVM, v.1.1.1)^45^. The gene models were finally updated through PASA to produce UTRs and alternative splicing variations. We generated 40,464 gene models in total. The gene annotation results were evaluated by identifying complete BUSCO hits (1,283, 89.1%) in the *A. chinensis* genome v3.0.

### Functional annotation

We annotated the functions of the protein-coding genes using BLASTP (*E*-value cut-off 1e−5)^46^ based on entries in the Swiss-Prot and TrEMBL databases^19^. Protein domains were determined by searching against InterPro^19^. We also used Blast2GO^47^ to determine functions and pathways according to the Gene Ontology (GO)^21^ and Kyoto Encyclopedia of Genes and Genomes (KEGG) database^22^. In total, 38,202 genes were annotated through reference to one or more databases (Supplementary Fig. 8; Supplementary Table 12).

### Genome evolution and expansion/contraction of gene families

To investigate the evolutionary history of the *A. chinensis* genome, we analyzed the relationships of its protein-coding genes with those of eight other plant species: *O. sativa*, *A. thaliana*, *C. roseus*, *C. canephora*, *S. lycopersicum*, *S. tuberosum*, *C. sinensis*, and *R. delavayi*. For this, we carried out an all-vs.-all comparison using BLASTP (v2.2.26)^46^ (*E*-value cut-off: 1 × 10^−5^) and then applied OrthoMCL^23^ to group all the BLASTP results into orthologous and paralogous clusters.

We used 1517 high-quality single-copy genes to establish a phylogenetic tree. First, we aligned the coded protein sequences of these orthologous genes using MAFFT^48^ and then filtered the alignments using Gblocks^49^ by removing poorly aligned regions. We constructed gene trees separately for each gene alignment by RAxML (v8.0.0)^50^ with 500 bootstrap replicates. Divergence times between the sampled species were estimated using MCMCTREE of the PAML package (v4)^24^ with ‘correlated molecular clock’ and ‘JC69’ model. The gene tree of the concatenated 1517 single-copy orthologs was first used as input for MCMCtree. We calibrated this tree using the estimated divergence times in the TimeTree database (http://www.timetree.org/) for (1) *S. tuberosum-C. roseus* (77–91 Ma), (2) *A. thaliana-O. sativa* (148–173 Ma), (3) *A. thaliana-C. sinensis* (110–124 Ma), and (4) *C. roseus-C. sinensis* (93–116 Ma).

We investigated the expansion and contraction of gene families by clustering all homologs in the nine species as determined by OrthoMCL and estimating their sizes using the CAFE package (v4.2) and the previously inferred single-copy gene tree input as the species tree^26^. Through this approach, we identified a total of 3766 and 1738 kiwifruit gene families that had apparently expanded and contracted, respectively. We identified functional annotations of these genes and performed enrichment analysis using Blast2GO^47^.

### Synteny and whole-genome duplication

We applied the MCScanX^28^ package with default parameters to search for syntenic blocks, defined as regions with more than five collinear genes, between paired genomes. We then calculated synonymous substitution rates (*Ks*) of the collinear orthologous gene pairs using the Perl script “add_ka_and_ks_to_collinearity.pl” implemented in the MCScanX package. The *Ks* values were converted to divergence times according to the formula *T* = *Ks*/2*r*, where *T* is divergence time and *r* is the neutral substitution rate (*r* = 3.39 × 10^−9^). We also used a previously published approach^10^ to extract homologous gene groups to construct phylogenetic trees of the orthologous genes. Finally, we identified likely nodes for whole-genome duplications based on the phylogenetic relationships of the homologous genes.

## Supplementary information

Supplementary Figure

Supplementary Table

Supplementary Table 8

## Data Availability

Raw sequencing reads used for de novo whole-genome assembly and the final genome have been deposited in the Sequence Read Archive database under accession number PRJNA549770.
