# Supplementary material for: A high-quality Actinidia chinensis (kiwifruit) genome
Source: Hortic Res. 2019 Oct 15;6:117. doi: 10.1038/s41438-019-0202-y (PMC6804796; doi:10.1038/s41438-019-0202-y)
Supplement: Supplementary file 1 — Supplementary Figure [file 41438_2019_202_MOESM1_ESM.docx]

**Supplementary Figures**


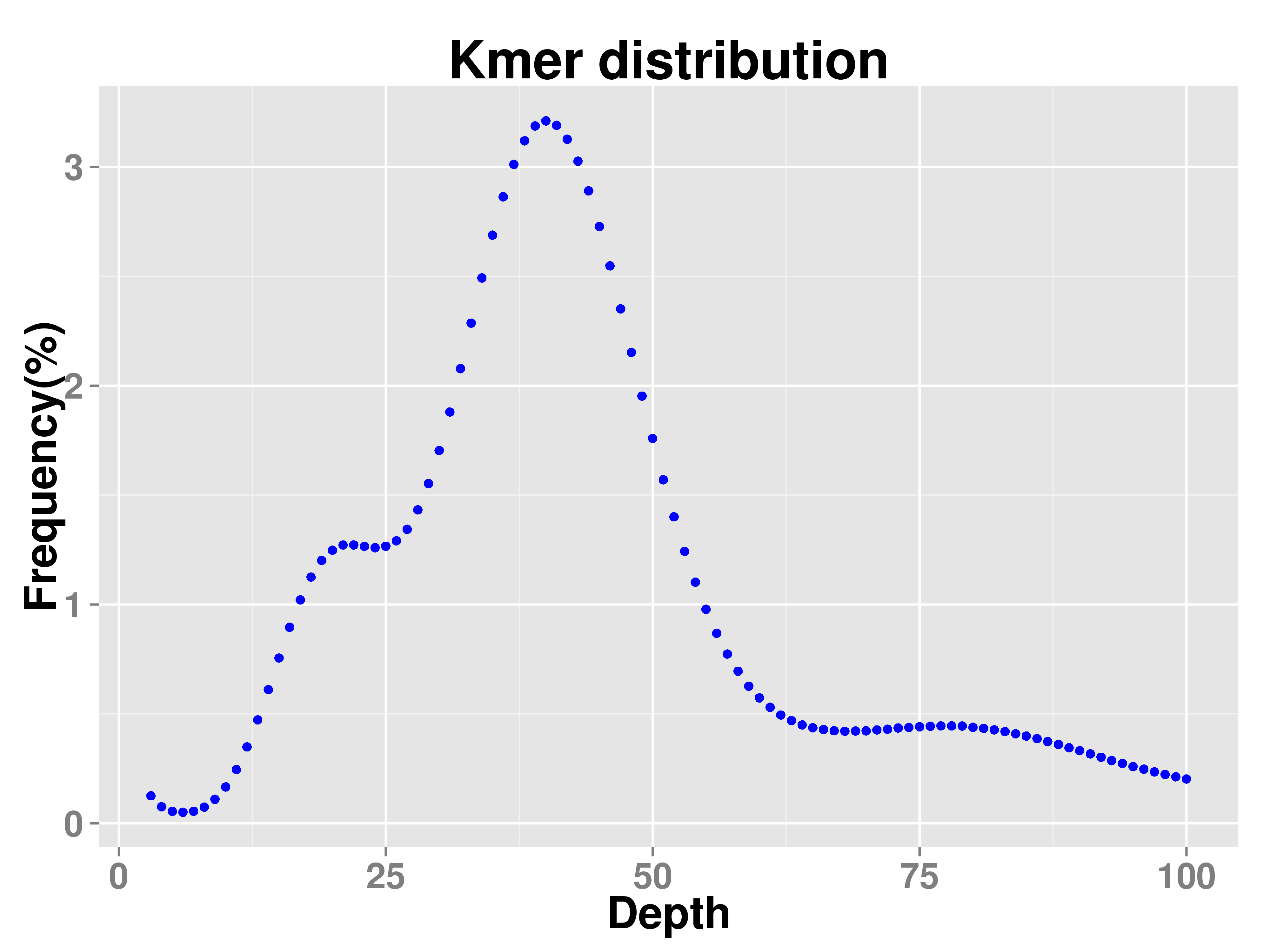


Supplementary Figure 1. k-mer distribution (19-mer) of the kiwifruit Illumina sequencing reads. The frequency of each 19-mer was calculated based on the filtered paired-end reads from libraries with short inserts (270bp). Two peaks were observed (at ~20x and 40x, respectively) indicating heterozygosity in “Hongyang”. The genome size was estimated to be 637.99 Mb.

Supplementary Figure 2. The pipeline for Hi-C directed chromosome assembly in this study. After quality assessment of Hi-C library, those valid interactions were used for genome assembly, including grouping, ordering and orienting scaffolds.


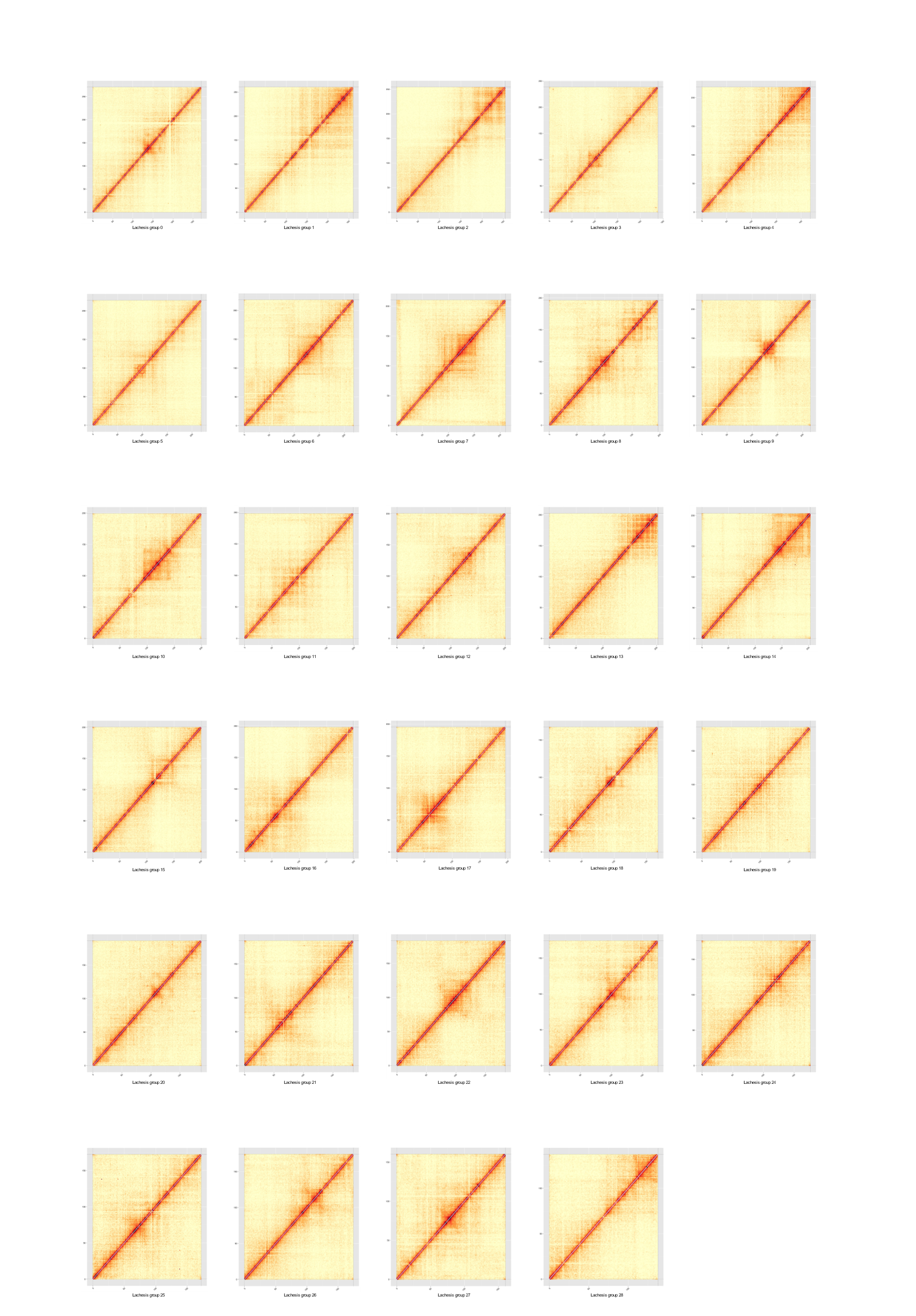


Supplementary Figure 3. Chromatin interactions in each chromosome of *A. chinensis*. Each heatmap is shown at a resolution of 200 kb. The dark red dots show high probability of interaction and light yellow show low probability of interaction.


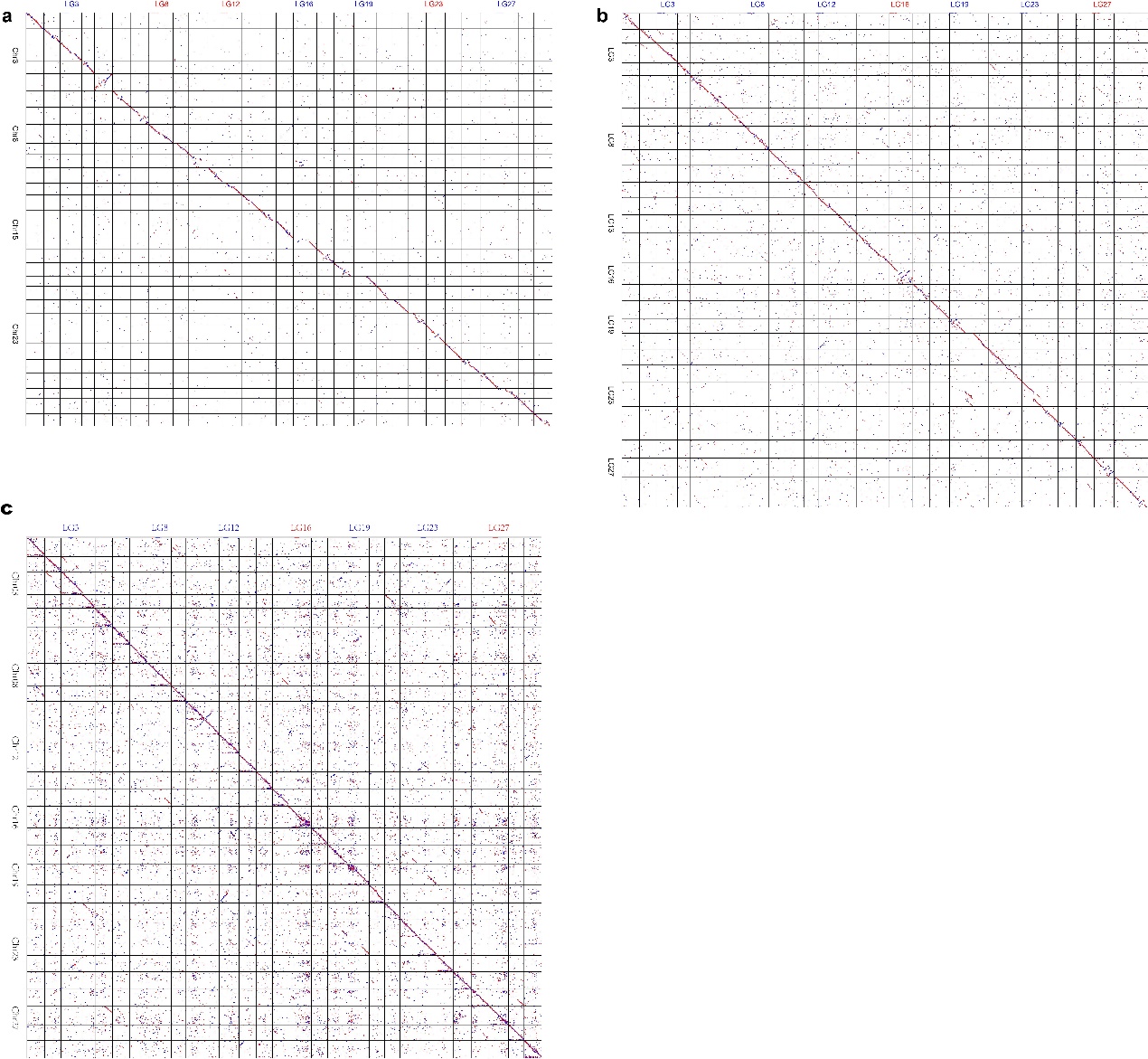


Supplementary Figure 4. lastdot-plot comparison of *A. chinensis* genome v3.0 with the v1.0 (a), the v2.0 (b) assembly and *A. eriantha* genome (c). *A. chinensis* genome v3.0 chromosomes (X-axis) were aligned to v1.0 chromosomes, v2.0 chromosomes and *A. eriantha* chromosomes (Y-axis) using LAST and alignments were subsequently filtered for 1-on-1 alignments. Chromosomal inversions and breakage are shown as blue lines.


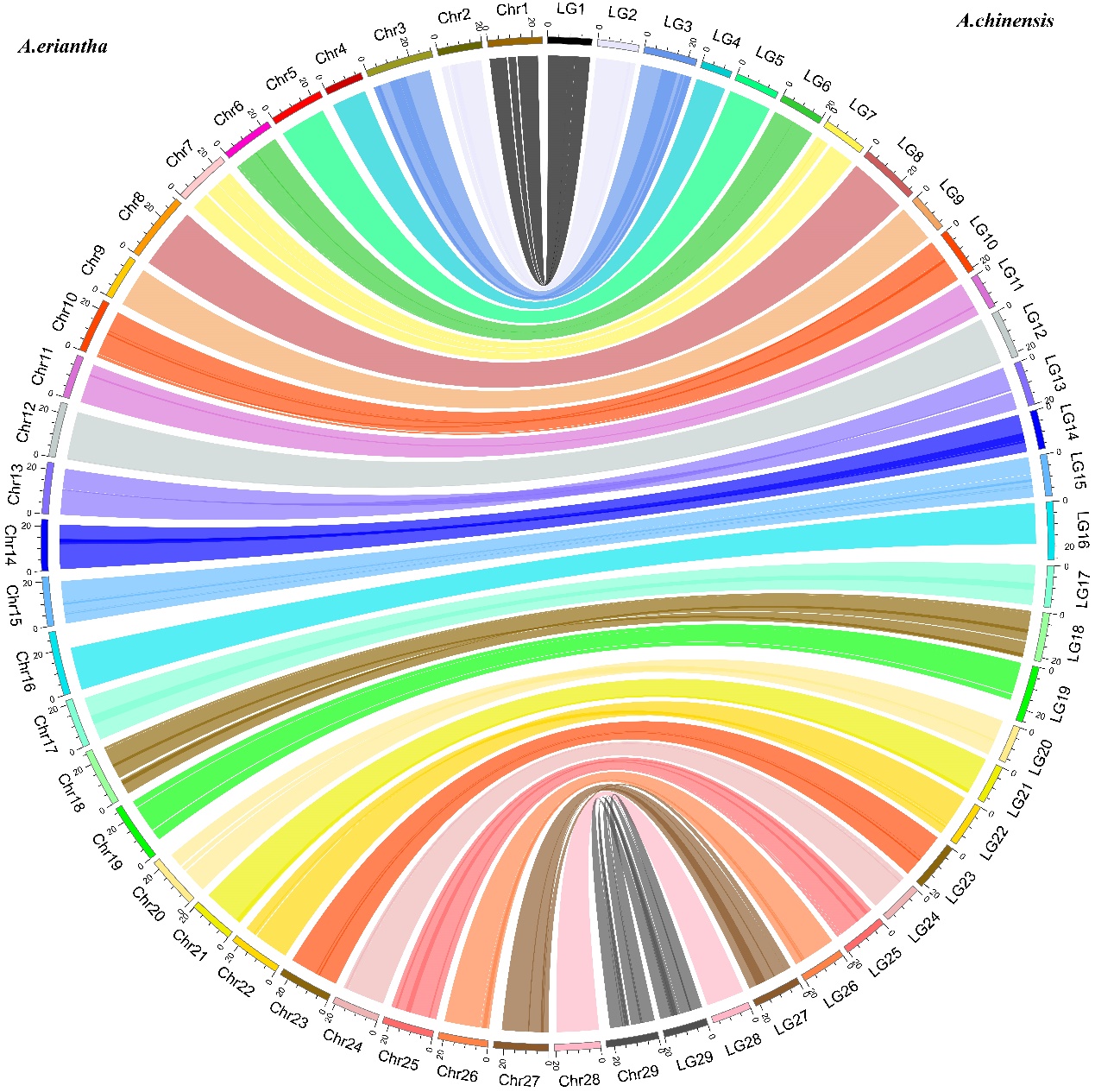


Supplementary Figure 5. The landscape of genome synteny between *A. chinensis* and *A. eriantha*.

Supplementary Figure 6. The statistics of the number of three types LTRs in the three *A. chinensis* genome versions.


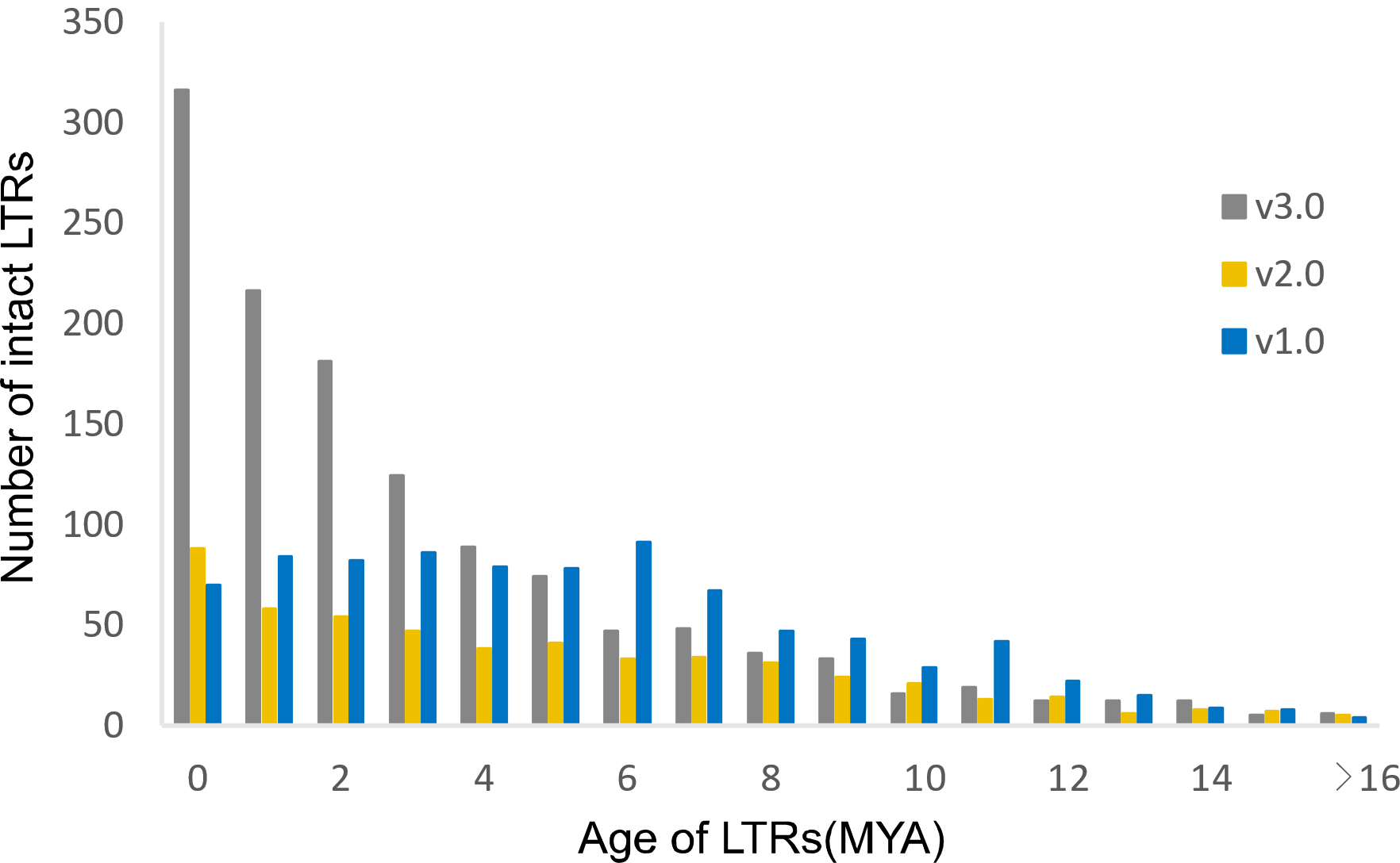


Supplementary Figure 7. The number of intact LTR-RTs birthed at different times (million years ago, MYA) in the three assemblies of the *A. chinensis* genome.

Supplementary Figure 8. Comparison of raw LAI scores among three *A. chinensis* genome versions using different techniques.

Supplementary Figure 9. The functional annotations of gene models by InterPro, Swissprot, TrEMBL, GO and KEGG Pathway databases.


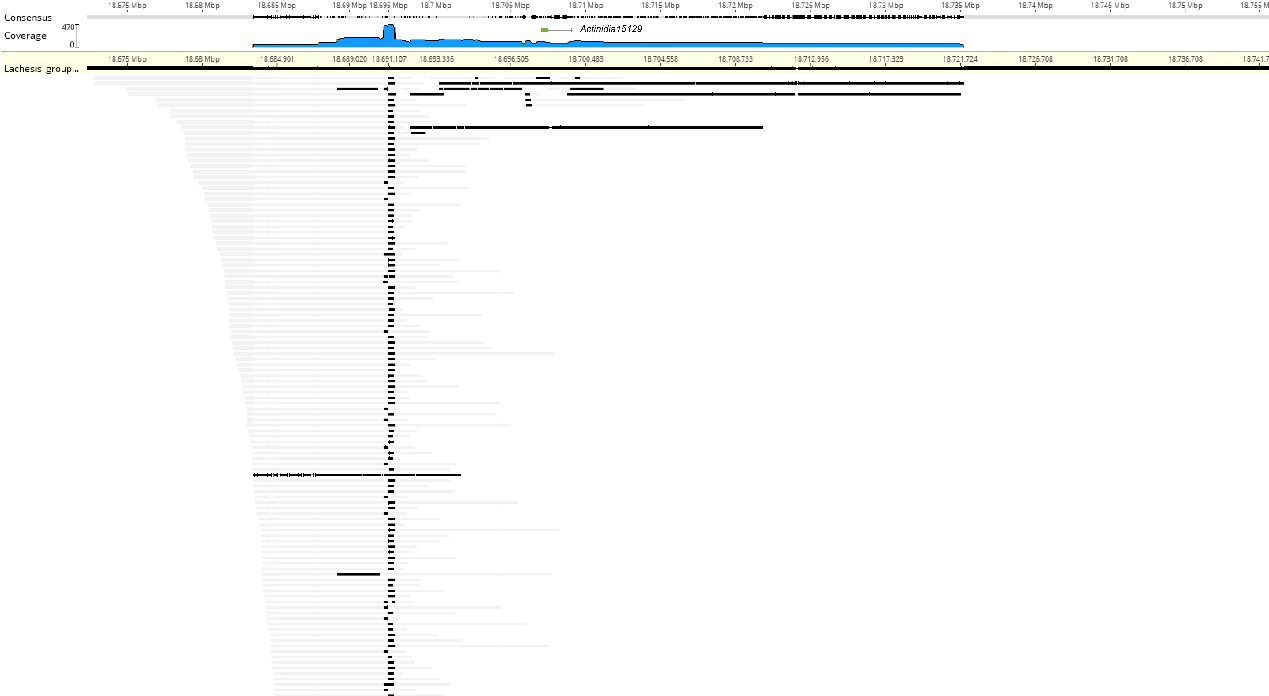


Supplementary Figure 10. Verifying the accuracy of *Actinidia15129* by mapping the PacBio reads to *A. chinensis* genome v3.0.


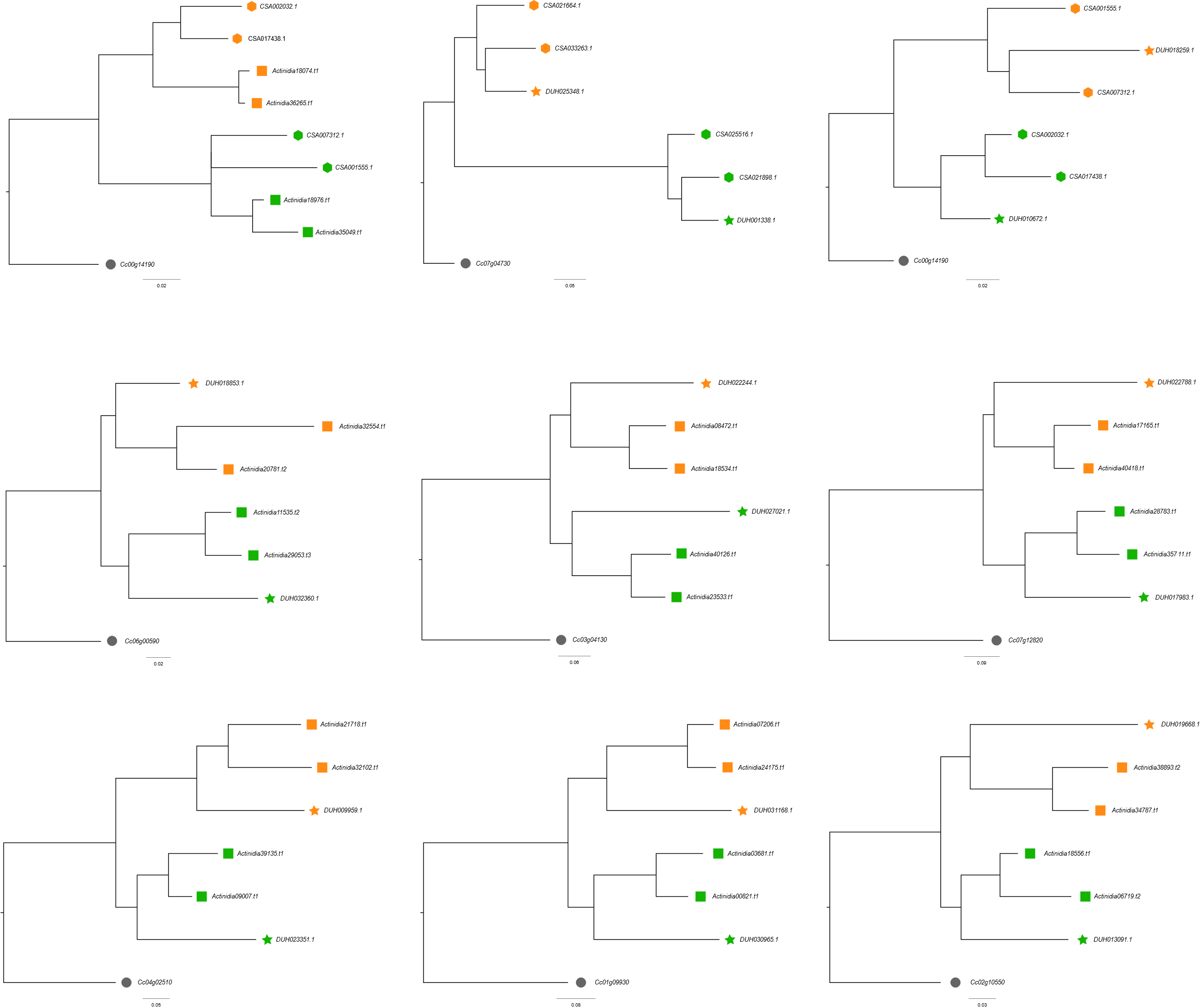


Supplementary Figure 11. Phylogenetic tree of homologous genes from *Actinidia* (rectangle), *Rhododendron* (star), *Camella* (hexagon) and *Coffea* (circle), supporting the shared A*d*-β event in Ericales. Homoeoloous groups related to the eudicot-common hexaploidy are shown with black color, and homologous genes belonged to A*d*-α and A*d*-β events were shown in orange and green color, respectively.
