# Supplementary material for: A high-quality Actinidia chinensis (kiwifruit) genome
Source: Hortic Res. 2019 Oct 15;6:117. doi: 10.1038/s41438-019-0202-y (PMC6804796; doi:10.1038/s41438-019-0202-y)
Supplement: Supplementary file 2 — Supplementary Table [file 41438_2019_202_MOESM2_ESM.docx]

**Supplementary table 1.** **Metrics of the different steps performed for genome assembly in** ***A. chinensis* genome v3.0**

|  | Contigs (PacBio) | Contigs (Hi-C corrected) | Scaffolds (Hi-C) |
| --- | --- | --- | --- |
| Number of sequences | 2,231 | 2,366 | 1720 |
| Total length (Mb) | 653.86 | 653.86 | 653.93 |
| N50 (Mb) | 1.72 | 1.43 | 20.0 |
| N90 (kb) | 166.4 | 127.9 | 264.7 |
| Longest | 10.0Mb | 7.8Mb | 27.3Mb |
| GC content | 35.63% | 35.63% | 35.63% |
| Repeat region % of assembly | 44.19% | 43.43% | 43.42% |

**Supplementary table 2. The statistics of Illumina reads mapped to *A. chinensis* genome v3.0**

| **Library** | **Total reads** | **Mapped reads** | **Mapped (%)** | | **Properly mapped reads** | **Properly mapped (%)** |
| --- | --- | --- | --- | --- | --- | --- |
| 270 bp | 204,986,879 | 202,307,143 | 98.69% | 186,150,446 | | 92.47% |

**Supplementary table 3. The RNA-seq data and mapping statistics in this study.**

| **SRA accession** | **Tissue** | **platform** | **Read type** | **Number of reads** | **Read length (bp)** | **Total data (G**b) | **Mapping efficiency (%)** |
| --- | --- | --- | --- | --- | --- | --- | --- |
| SRR5650770 | Flowers | Illumina | PE | 53,550,000 | 2 × 100 | 10.7 | 86.72 |
| SRR653044 | Leaves | Illumina | PE | 26,608,222 | 2 × 90 | 4.8 | 90.83 |
| SRR653045 | Fruits (20d) | Illumina | PE | 27,462,864 | 2 × 90 | 4.9 | 93.48 |
| SRR653046 | Fruits (120d) | Illumina | PE | 26,655,278 | 2 × 90 | 4.8 | 89.67 |
| SRR653047 | Fruits (127d) | Illumina | PE | 27,012,822 | 2 × 90 | 4.9 | 89.78 |
| / | Leaves | PacBio | / | 53,054 | / | 0.1 | 95.53 |

**Supplementary table 4. Quality assessment of the assembled genome of *A. chinensis* using CEMGA.**

| **Species** | **Number of 458 CEGs* present in assembly** | **% of 458 CEGs present in assemblies** | **Number of 248 highly conserved CEGs present** | **% of 248 highly conserved CEGs present** |
| --- | --- | --- | --- | --- |
| *Actinidia Chinensis* | 427 | 93.23% | 197 | 79.44% |

**Supplementary table 5. Quality assessment of the assembled genome of** ***A. chinensis* using BUSCOs.**

| **Type** | **Number** | **Percent (%)** |
| --- | --- | --- |
| Complete BUSCOs (C) | 1,307 | 90.8 |
| Complete and single-copy BUSCOs (S) | 1,083 | 75.2 |
| Complete and duplicated BUSCOs (D) | 224 | 15.6 |
| Fragmented BUSCOs (F) | 31 | 2.2 |
| Missing BUSCOs (M) | 102 | 7.0 |
| Total BUSCO groups searched | 1,440 | 100 |

**Supplementary Table 6. The mapping statistics of in vivo Hi-C libraries. Totally, ~226.4 million paired-end reads were generated which covered ~116**× **of *A. chinensis* genome. Only unique paired alignments (~141.5 million) were used for downstream analysis, and the valid interaction pairs (~104.4 million) were used to build the interaction matrices**.

| **Type** | **Number** | **Ratio(%)** |
| --- | --- | --- |
| Total Read Pairs | 253,665,959 | 100 |
| Mapped Reads | 418,274,258 | 82.45 |
| Unique Paired Alignments | 141,478,565 | 55.77 |
| Valid Interaction Pairs | 104,411,284 | 41.16 |
| Dangling End Pairs | 15,006,799 | 5.92 |
| Re-ligation Pairs | 2,195,274 | 0.87 |
| Self-cycle Pairs | 6,269,749 | 2.47 |
| Dumped Pairs | 13,595,459 | 5.36 |

**Supplementary Table 7. Detailed pseudochromosome relationship of three *A. chinensis* genome assembly.**

| Before change pseudomolecules name | | | After change pseudomolecules name | | |
| --- | --- | --- | --- | --- | --- |
| v1.0 | v2.0 | v3.0 | v1.0 | v2.0 | v3.0 |
| Chr1 | CM009654.1 | Lachesis Group8 | Chr1 | LG1 | LG1 |
| Chr2 | CM009655.1 | Lachesis Group21 | Chr2 | LG2 | LG2 |
| Chr3 | CM009656.1 | Lachesis Group3 | Chr3 | LG3 | LG3 |
| Chr4 | CM009657.1 | Lachesis Group28 | Chr4 | LG4 | LG4 |
| Chr5 | CM009658.1 | Lachesis Group11 | Chr5 | LG5 | LG5 |
| Chr6 | CM009659.1 | Lachesis Group12 | Chr6 | LG6 | LG6 |
| Chr7 | CM009660.1 | Lachesis Group16 | Chr7 | LG7 | LG7 |
| Chr8 | CM009661.1 | Lachesis Group0 | Chr8 | LG8 | LG8 |
| Chr9 | CM009662.1 | Lachesis Group25 | Chr9 | LG9 | LG9 |
| Chr10 | CM009663.1 | Lachesis Group7 | Chr10 | LG10 | LG10 |
| Chr11 | CM009664.1 | Lachesis Group27 | Chr11 | LG11 | LG11 |
| Chr12 | CM009665.1 | Lachesis Group5 | Chr12 | LG12 | LG12 |
| Chr13 | CM009666.1 | Lachesis Group15 | Chr13 | LG13 | LG13 |
| Chr14 | CM009667.1 | Lachesis Group23 | Chr14 | LG14 | LG14 |
| Chr15 | CM009668.1 | Lachesis Group20 | Chr15 | LG15 | LG15 |
| Chr16 | CM009669.1 | Lachesis Group1 | Chr16 | LG16 | LG16 |
| Chr17 | CM009670.1 | Lachesis Group19 | Chr17 | LG17 | LG17 |
| Chr18 | CM009671.1 | Lachesis Group6 | Chr18 | LG18 | LG18 |
| Chr19 | CM009672.1 | Lachesis Group2 | Chr19 | LG19 | LG19 |
| Chr20 | CM009673.1 | Lachesis Group18 | Chr20 | LG20 | LG20 |
| Chr21 | CM009674.1 | Lachesis Group26 | Chr21 | LG21 | LG21 |
| Chr22 | CM009675.1 | Lachesis Group17 | Chr22 | LG22 | LG22 |
| Chr23 | CM009676.1 | Lachesis Group9 | Chr23 | LG23 | LG23 |
| Chr24 | CM009677.1 | Lachesis Group22 | Chr24 | LG24 | LG24 |
| Chr25 | CM009678.1 | Lachesis Group14 | Chr25 | LG25 | LG25 |
| Chr26 | CM009679.1 | Lachesis Group13 | Chr26 | LG26 | LG26 |
| Chr27 | CM009680.1 | Lachesis Group4 | Chr27 | LG27 | LG27 |
| Chr28 | CM009681.1 | Lachesis Group24 | Chr28 | LG28 | LG28 |
| Chr29 | CM009682.1 | Lachesis Group10 | Chr29 | LG29 | LG29 |

**Supplementary Table 8. Detailed information of each chromosome for corresponding version genome of *A. chinensis*.**

This table is provided as Microsoft Excel spreadsheet.

Sheet 1 (gene_number_on_chr): statistics of gene number on each chromosome for corresponding version genome;

Sheet 2 (chr_length): statistics of each chromosome length for corresponding version genome;

Sheet 3 (gap_in_chr): statistics of gap number on each chromosome for corresponding version genome;

Sheet 4 (gap_length_in_chr): statistics of gap length of each chromosome for corresponding version genome;

**Supplementary Table 9. The statistics of gaps in three *A. chinensis* genome assembly.**

|  | v1.0 | v2.0 | v3.0 |
| --- | --- | --- | --- |
| total gaps number | 26,775 | 25,521 | 646 |
| gaps number per Mb | 43.46 | 46.36 | 0.98 |
| total gaps length | 27,160,880 | 19,766,770 | 64,600 |
| gaps length per Mb | 44084.47 | 35906.94 | 98.30 |

**Supplementary Table 10. The statistics of LTRs in three *A. chinensis* genome assembly.**

|  | v1.0 | v2.0 | v3.0 |
| --- | --- | --- | --- |
| Number of LTR elements | 346,220 | 233,273 | 250,325 |
| Length of LTR elements | 152,277,870 | 102,060,066 | 152,863,036 |
| Percentage of total sequence | 24.12% | 18.43% | 23.38% |

**Supplementary Table 11. Summary of intact LTR-RTS in three *A. chinensis* assemblies.**

|  | v1.0 | v2.0 | v3.0 |
| --- | --- | --- | --- |
| *Copia* | 237 | 171 | 333 |
| *Gypsy* | 240 | 115 | 429 |
| Unknown | 379 | 236 | 484 |
| Total on chromosome | 620 | 521 | 1212 |
| Total | 856 | 522 | 1,246 |

**Supplementary Table 12. Summary of the functional annotation in *A. chinensis* genome v3.0.**

| **Annotation database** | **Annotated number** | **Percentage (%)** |
| --- | --- | --- |
| Swissprot | 28,934 | 71.51% |
| TrEMBL | 34,639 | 85.60% |
| InterPro | 37,855 | 93.55% |
| GO | 33,108 | 81.82% |
| KEGG Pathway | 9,855 | 24.35% |
| Total | 38,202 | 94.41% |

**Supplementary Table 13. Comparison of genome annotations by BUSCO analysis of the new assemblies and the previously published assemblies of *A. chinensis* genomes.**

|  | v1.0 | | v2.0 | | v3.0 | |
| --- | --- | --- | --- | --- | --- | --- |
|  | number | Percent (%) | number | Percent (%) | number | Percent (%) |
| Complete BUSCOs (C) | 1102 | 76.6 | 1361 | 94.5 | 1283 | 89.1 |
| Complete and single-copy BUSCOs (S) | 836 | 58.1 | 996 | 69.2 | 1068 | 74.2 |
| Complete and duplicated BUSCOs (D) | 266 | 18.5 | 365 | 25.3 | 215 | 14.9 |
| Fragmented BUSCOs (F) | 153 | 10.6 | 34 | 2.4 | 66 | 4.6 |
| Missing BUSCOs (M) | 185 | 12.8 | 45 | 3.1 | 91 | 6.3 |
| Total BUSCO group searched | 1440 | 100 | 1440 | 100 | 1440 | 100 |

**Supplementary Table 14. Statistics of predicted gene models in three *A. chinensis* genome.**

|  | v1.0 | v2.0 | v3.0 |
| --- | --- | --- | --- |
| Gene on chromosome | 31,409 | 32,951 | 36,482 |
| gene not on chromosome | 8,352 | 93 | 3,982 |
| Gene with gap | 2,779 | 4,365 | 0 |
| version-specific gene | 2,205 | 575 | 2,750 |
| Total gene | 39,761 | 33,044 | 40,464 |
| average CDS length | 1,103bp | 1,047bp | 1,028bp |
| exon per gene | 4.49 | 4.82 | 5.47 |

**Supplementary Table 15. Summary of gene family clustering in *A. chinensis*.**

| **Species** | **Total**  **genes** | **Genes in families** | **Family** | **Unclustered genes** | **Unique families** | **Genes per family** |
| --- | --- | --- | --- | --- | --- | --- |
| *O.sativa* | 39049 | 26492 | 13916 | 12557 | 2338 | 1.90 |
| *A.thaliana* | 27416 | 22402 | 13903 | 5014 | 932 | 1.61 |
| *C.roseus* | 33829 | 28066 | 14562 | 5763 | 741 | 1.93 |
| *C.canephora* | 25574 | 20066 | 15109 | 5508 | 603 | 1.33 |
| *S.lycopersicum* | 34725 | 24958 | 18454 | 9767 | 550 | 1.35 |
| *S.tuberosum* | 39028 | 30256 | 17941 | 8772 | 868 | 1.69 |
| *C.sinensis* | 36951 | 22010 | 14599 | 14941 | 825 | 1.51 |
| *R.delavayi* | 32938 | 24246 | 15735 | 8692 | 1141 | 1.54 |
| *A.chinensis* | 40464 | 23560 | 15294 | 16904 | 875 | 1.54 |

**Supplementary Table 16. Summary of three whole-genome duplication and speciation events in *A. chinensis*.**

|  | *Ks* peak value | MYA |
| --- | --- | --- |
| A*d*-α | 0.12-0.18 | 17.7-26.5 |
| A*d*-β | 0.42-0.50 | 61.9-73.7 |
| A*t*-γ | 1.02-1.08 | 150.4-159.3 |
| Speciation | 0.4 | 59.0 |
