# Supplementary material for: A high-quality Actinidia chinensis (kiwifruit) genome
Source: Hortic Res. 2019 Oct 15;6:117. doi: 10.1038/s41438-019-0202-y (PMC6804796; doi:10.1038/s41438-019-0202-y)
Supplement: Supplementary file 3 — Supplementary Table 8 [file 41438_2019_202_MOESM3_ESM.pdf]

	gene number		
chr	v1.0	v2.0	v3.0
LG1	1,150	1,111	1,208
LG2	1,153	1,089	1,247
LG3	1,742	1,723	1,893
LG4	853	771	807
LG5	1,205	950	1,148
LG6	1,323	1,049	1,353
LG7	1,223	1,065	1,184
LG8	1,437	1,439	1,612
LG9	811	1,112	1,173
LG10	648	979	1,076
LG11	1,124	1,104	1,043
LG12	778	1,049	1,225
LG13	1,404	1,384	1,586
LG14	719	1,100	1,075
LG15	1,417	1,100	1,362
LG16	813	1,245	1,184
LG17	983	924	1,113
LG18	1,018	1,210	1,295
LG19	1,014	646	1,521
LG20	922	1,050	1,154
LG21	1,013	1,039	1,132
LG22	729	1,079	1,122
LG23	1,807	2,316	1,850
LG24	1,250	1,190	1,287
LG25	835	997	1,085
LG26	1,236	1,230	1,315
LG27	739	1,008	1,067
LG28	1,165	1,008	1,239
LG29	898	984	1,126
total	31,409	32,951	36,482

	chr_length		
chr	v1.0	v2.0	v3.0
LG1	17,971,566	18,577,340	22,844,385
LG2	15,003,700	14,622,108	20,054,939
LG3	20,519,224	21,733,881	26,632,065
LG4	13,065,296	13,767,589	15,755,053
LG5	18,825,850	18,594,566	22,454,775
LG6	18,208,465	17,379,730	22,059,169
LG7	18,704,015	20,027,516	21,071,456
LG8	21,083,321	26,073,903	29,586,325
LG9	11,598,160	16,576,380	18,930,973
LG10	15,490,421	19,347,361	23,534,242
LG11	17,082,262	16,878,413	17,372,815
LG12	13,002,763	19,193,532	23,906,174
LG13	17,372,821	19,520,183	21,416,912
LG14	12,031,045	17,889,010	20,002,199
LG15	19,001,909	15,946,168	20,197,523
LG16	11,476,499	23,784,918	29,254,477
LG17	14,675,105	17,432,628	20,244,552
LG18	14,814,751	20,725,234	23,439,541
LG19	11,145,676	15,405,244	27,852,454
LG20	14,375,345	17,941,880	21,063,375
LG21	14,287,830	17,317,561	18,637,856
LG22	12,982,530	18,944,233	21,314,356
LG23	20,688,064	27,689,248	22,761,933
LG24	17,702,303	17,819,793	19,944,602
LG25	14,321,294	19,628,047	21,886,988
LG26	17,153,587	20,385,717	22,033,470
LG27	10,917,718	20,970,093	24,132,663
LG28	16,406,049	15,843,535	19,677,420
LG29	14,180,785	17,963,318	22,689,326
Total	#########	547,979,129	640,752,018

	gap number		
chr	v1.0	v2.0	v3.0
LG1	598	845	18
LG2	527	691	21
LG3	634	952	28
LG4	476	690	14
LG5	664	904	22
LG6	616	836	20
LG7	414	871	23
LG8	707	1177	36
LG9	308	794	23
LG10	553	965	19
LG11	521	782	20
LG12	434	966	25
LG13	517	873	22
LG14	438	752	23
LG15	614	638	17
LG16	306	1164	37
LG17	554	867	28
LG18	461	925	16
LG19	254	794	26
LG20	512	822	23
LG21	454	752	13
LG22	404	876	26
LG23	556	1145	21
LG24	456	745	15
LG25	425	862	20
LG26	516	902	21
LG27	319	1048	15
LG28	473	732	23
LG29	452	940	31
total	14,163	25,310	646

	gap length		
chr	v1.0	v2.0	v3.0
LG1	402,756	572,775	1,800
LG2	326,569	456,395	2,100
LG3	337,397	647,925	2,800
LG4	296,778	558,395	1,400
LG5	443,938	837,400	2,200
LG6	405,868	652,525	2,000
LG7	283,282	603,177	2,300
LG8	374,018	899,627	3,600
LG9	183,434	539,907	2,300
LG10	487,105	892,213	1,900
LG11	368,121	539,806	2,000
LG12	299,177	747,918	2,500
LG13	277,445	603,346	2,200
LG14	282,370	608,039	2,300
LG15	385,679	486,099	1,700
LG16	189,434	1,131,544	3,700
LG17	314,541	730,533	2,800
LG18	299,462	881,775	1,600
LG19	164,551	824,783	2,600
LG20	270,392	490,999	2,300
LG21	247,467	629,834	1,300
LG22	297,252	606,350	2,600
LG23	279,431	619,518	2,100
LG24	237,165	468,682	1,500
LG25	302,812	584,808	2,000
LG26	338,507	710,700	2,100
LG27	231,242	994,302	1,500
LG28	334,982	497,422	2,300
LG29	294,034	735,181	3,100
total	8,955,209	19,551,978	64,600
